# The Metaverse, the Built Environment, and Public Health: Opportunities and Uncertainties

**DOI:** 10.2196/43549

**Published:** 2023-02-13

**Authors:** Mohammad Javad Koohsari, Gavin R McCormack, Tomoki Nakaya, Akitomo Yasunaga, Daniel Fuller, Yukari Nagai, Koichiro Oka

**Affiliations:** 1 School of Knowledge Science Japan Advanced Institute of Science and Technology Nomi Japan; 2 Faculty of Sport Sciences Waseda University Tokorozawa Japan; 3 Department of Community Health Sciences Cumming School of Medicine University of Calgary Calgary, AB Canada; 4 Faculty of Kinesiology University of Calgary Calgary, AB Canada; 5 School of Architecture, Planning and Landscape University of Calgary Calgary, AB Canada; 6 Graduate School of Environmental Studies Tohoku University Sendai Japan; 7 Faculty of Liberal Arts and Sciences Bunka Gakuen University Tokyo Japan; 8 Department of Community Health and Epidemiology College of Medicine University of Saskatchewan Saskatchewan, SK Canada

**Keywords:** virtual reality, technology, neighborhood, urban design, health, epidemiology, artificial intelligence, sport sciences, augmented reality, health care

## Abstract

There has been a growing interest in the “metaverse,” and discourse about how this platform may contribute to different fields of science is already beginning to emerge. In this paper, we discuss key opportunities and uncertainties about how a metaverse might contribute to advancing knowledge in the interdisciplinary field of the built environment and public health aimed at reducing noncommunicable diseases.

## Introduction

Since 2021, there has been a growing interest in the “metaverse,” and discourse about how this platform will contribute to different fields of science is already beginning to emerge [1-5]. However, scientific discussions regarding the potential research applications of the metaverse are still in their infancy. A PubMed search of published literature, including the keyword “metaverse,” undertaken in July 2022, revealed only 58 articles, of which 49 were published in 2022. Of these, several viewpoint articles discussed how a metaverse could contribute to different aspects of health, such as medicine [5], cardiovascular health [2,4], dentistry [5], ophthalmology [6], and intelligence health care [7]. Nevertheless, none of these previous viewpoint articles focused on the “built environment” and health. The built environment can be defined as “the human-made [physical] space in which people live, work, and recreate on a day-to-day basis” [8], such as houses, shops, roads, parks, and public spaces. The built environment can potentially impact health over the long-term via its influences on health behaviors [9,10]. As a first study, to our knowledge, the purpose of this paper is to discuss key opportunities and uncertainties about how a metaverse might contribute to advancing knowledge in the interdisciplinary field of the built environment and public health aimed at reducing noncommunicable diseases. The rationale for selecting noncommunicable diseases as a topic of focus is that these diseases (eg, diabetes, heart diseases, strokes, chronic respiratory disease, cancers, and mental illness) are potentially avoidable but remain the primary causes of deaths worldwide, accounting for 74% of all deaths in 2019 [11]. As life expectancy increases, the burden of noncommunicable diseases will continue to increase, especially in aging societies, such as Japan. Modifiable risk factors that can lead to noncommunicable diseases (eg, physical inactivity, unhealthy diet, and smoking) are prevalent in many populations. The built environment plays a pivotal role in shaping these modifiable risk factors. Population-level interventions affecting many people over a relatively long time that reduce these risk factors are necessary to manage the increase in noncommunicable diseases [12]. This paper will be of interest to experts in public health, urban design, epidemiology, medicine, sport, and environmental sciences, especially those considering using the metaverse for research and intervention purposes.

## Existing Knowledge of the Relationships Between the Built Environment and Noncommunicable Diseases

Modifying the built environment is an essential population-level strategy for changing noncommunicable disease risk factors [10]. Several systematic reviews have provided evidence on the relationships between built environment attributes and noncommunicable disease risk factors [13,14]. Several pathways through which the built environment may influence noncommunicable diseases have also been discussed in previous studies [15,16]. For example, Koohsari et al [15] proposed a conceptual framework by which built environment characteristics impact cardiovascular diseases via behavioral (eg, physical activity, sedentary behavior, diet, sleep), ecological (eg, air/noise pollution and heat), and physiological risk factors.

## What Is a Metaverse?

In 1992, the term “metaverse” first appeared in Neil Stevenson’s science fiction novel, *Snow Crash*, where people relate with each other through avatars in a virtual world. The term has recently regained considerable attention after Facebook announced the change of their name to “Meta” in October 2021 as a metaverse company. Since then, academics from several scientific disciplines have developed definitions of a metaverse and proposed research opportunities that it may offer.

Since many disciplines are involved in developing a metaverse, reaching a universally accepted definition of this term may be challenging. Therefore, there is yet to be a consensus on the definition of a metaverse. In the hospitality and tourism field, Gursoy et al [3] defined a metaverse as “a parallel reality where humans can work, play, and communicate.” In a more comprehensive definition, a metaverse is “a three-dimensional virtual world where avatars engage in political, economic, social, and cultural activities” [17]. In the field of medicine, Yang et al [1] defined a metaverse as “the internet accessed via virtual reality and augmented reality glasses.” In relation to cardiovascular health, Mesko [4] referred to a metaverse as “a virtual reality space in which users can interact with other users in a computer-generated social environment.” Several technologies such as virtual reality, augmented reality, and artificial intelligence are necessary elements in building a metaverse. However, a broader definition is needed as the technologies that facilitate how people interact in a metaverse will change over time.

## Opportunities and Uncertainties

Knowledge of how the built environment may influence noncommunicable diseases has improved over the past decade. However, there are a number of overarching limitations to this research, which may be enhanced by conducting research in a metaverse.

### Opportunities

#### Conducting Randomized Experimental Studies

Because of ethical and budgetary constraints, there is a lack of randomized experimental study designs examining the causal effects of the built environment on noncommunicable disease and health more broadly [18,19]. Two theoretical concepts of *immersion* and *presence* in a metaverse can potentially facilitate conducting these experiments. In a metaverse, the concept of immersion is defined as “users’ engagement with a virtual reality system that results with being in a flow state” [20]. A more subjective concept is that of presence, which refers to a feeling and sense of “being there” in a mediated and virtual world [21,22]. Within a metaverse, study participants could be randomized to experience different built environment exposures such as high and low density, high and low walkability, or different levels of nature or urban environments. A better understanding of how experiences of these virtual built environmental exposures affect people’s biomarkers, physiological and psychological responses, and health behavior decision-making in the short-term and noncommunicable diseases over the long-term could be tested in a metaverse [23]. It should be noted that if participants are required to experience certain environmental conditions virtually, such as extremely population-dense, loud, and overwhelming built environments in a metaverse, these experiences could potentially change attitudes or perceptions toward their environments, in reality, leading to changes in behavior [24]. Additionally, a metaverse can provide an opportunity to test how the built environment and social interactions contribute to people’s health. For instance, whether social interactions in a virtual park, reproduced from the real-world model, may contribute to health.

#### Offsetting or Alleviating the Effects of a Poor-Quality Built Environment

The experiments may reveal that interventions exposing individuals to supportive health environments in a metaverse positively impact psychological responses and behaviors in the physical world. For example, exposure to nature and green space in a metaverse may encourage individuals to seek out and use such spaces in the physical world. There may be political, financial, and logistical challenges to modifying built environment attributes such as street layouts, land uses, sidewalks, and green spaces. Changes to the built environment also often take a long time to implement. In some cases, modifying the built environment (eg, creating a new park) may be impossible, especially in densely built-up and populated areas lacking free spaces. In these situations, a metaverse may alleviate health problems by enabling people to experience a modified improved version of their physical environments virtually and feel a presence in those places. These virtual places may also provide opportunities for social interactions among avatars. For instance, workers in workplaces lacking green spaces can immerse themselves in greenery using a metaverse, which may offer mental health benefits. Alternatively, suppose a worker works from a virtual office within a metaverse. In that case, they could situate their virtual office anywhere—a café, warm cabin, mountain views, or the moon, which may alleviate stress. Based on the Yerkes-Dodson law, an individual’s performance improves up to a certain point with increasing mental arousal [25]. Such virtual offices can provide opportunities for workers to reach their optimal levels of positive arousal, which can promote their work performance. Nevertheless, at this stage, there is a lack of empirical studies examining the effects of exposing people to a health-supportive built environment in a metaverse on their health outcomes in the real world. Therefore, these opportunities remain to be confirmed or refuted by future studies.

#### Participation in Healthy Built Environment Design Interventions

**Figure 1:**
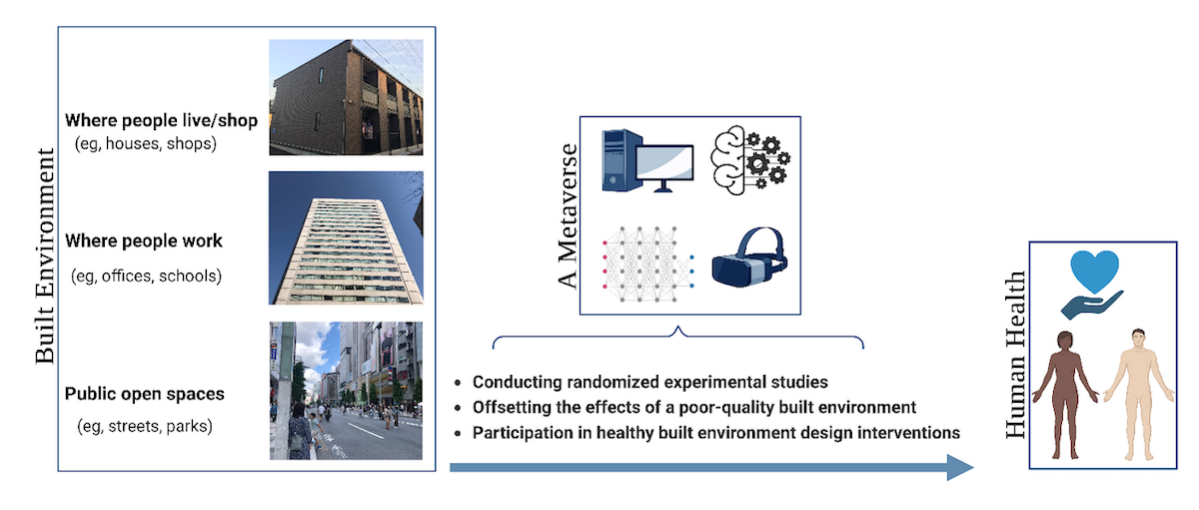
The built environment and human health: the opportunities offered by a metaverse (Part of the figure was created with BioRender.com).

Involving various stakeholders in the planning and design of health-support built environment interventions is necessary. Aligned with the immersion and presence concepts, a metaverse could allow stakeholders to experience, build, and collaboratively modify the proposed changes to the built environment before these interventions are implemented in the physical world. The application of virtual reality and its related technologies in participatory urban planning has already been discussed [26]. Additionally, with sufficient advances in health impact assessment, a metaverse could produce estimates of the health impacts of potential changes in real time. This allows urban designers and public health practitioners to optimize designs based on stakeholder feedback, social and cultural norms, health, socioeconomic, and inclusivity considerations (Figure 1).

### Uncertainties in the Impacts of a Metaverse on Health Behavior in the Physical World

At the time of writing this paper, there may also be some uncertainties about using a metaverse for research in the built environment and public health interdisciplinary field. Notable, how a metaverse impacts human behavior in the physical world will be a critical ongoing question. First, it is likely that not all the existing issues in the relationships between built environment and health can be discussed and tested in a metaverse in its current form. For instance, replicating the traditional research on the role of walkability on walking behavior may be challenging to undertake in a metaverse. It is then necessary to explore and identify the *senses* in the real world, which can be experienced in a metaverse and how different people can immerse themselves to those virtual environments. Second, a metaverse is a virtual manifestation and way to interact with people in a virtual world. It is uncertain but likely that current social structures may be recreated in a metaverse, particularly owing to the digital divide, defined as disparities between people due to their digital access, skills, and knowledge [27]. Thus, certain populations, including low-income or less-educated individuals and minority groups, may be excluded from a metaverse (or studies/research involving a metaverse) due to their lack of access, computer literacy, or feelings of safety to participate in research in a metaverse. Therefore, new ethical concerns about avoiding the digital divide may need to be addressed in relation to the research involving a metaverse [28]. Third, if individuals undertake much of their daily activities (and time) in a metaverse, they may isolate themselves from the physical world in favor of a metaverse, causing mental health consequences such as social isolation, depression, and antisocial behaviors. Notably, the social isolation triggered by online environments is an important consideration, especially in children and adolescents [29-31]. For example, an increase in online gaming was found to be associated with more minor adolescents’ social circles [31]. Fourth, this adoption of a “metaverse lifestyle” could also have potential unintentional physical health consequences by increasing physical inactivity. For instance, spending several hours per day in a metaverse could accumulate too much sitting time, which has been found to have several adverse physical health effects [32,33]. To avoid too much sitting, interacting in the metaverse should require physical movement in the real world. Active interaction in the virtual world can also promote exercise and physical activity. For instance, a review of randomized controlled trials found that engaging in active video games was beneficial for physical activity in adolescents [34]. Fifth, some of the most common acute health concerns experienced by patients using virtual environments are headaches, eye strain, vertigo, and nausea [35]. These health symptoms may negatively affect people’s physical function and further discourage them from engaging in healthy behaviors such as exercise and walking. Sixth, there may also be a reluctance to engage in urban design or community health decision-making because the physical world may seem less important to immersed people as the popularity of the metaverse increases. Finally, there are some concerns about the vital roles of technologies in creating a metaverse as a virtual space where we work, play, and communicate. Specifically, artificial intelligence (mimicking human intelligence in computers) technologies will be used for many functions in a metaverse. Artificial intelligence has been shown to recreate social patterns in multiple areas [36,37]. A metaverse relying heavily on artificial intelligence may recreate existing social patterns, including inequitable, ageist, racist, and heteronormative virtual environments. Considering these critical questions, recommendations or guidelines for healthy and safe use of a metaverse may need to be developed and promoted.

## Conclusion

A metaverse is evolving rapidly and has the potential to influence all aspects of human life. It is then best, sooner rather than later, to face the prospects and challenges a metaverse can offer to different scientific fields. Multidisciplinary research examining the impacts of the built environment on noncommunicable diseases in a metaverse will require our discipline to advance rapidly in many scientific areas. Thus, the current generation of the built environment and public health researchers will need to be versed in computer-assisted design and programming for artificial intelligence. They will need to work collaboratively with other disciplines (eg, information technology, software engineering, computer science) to use a metaverse to advance evidence-based knowledge on the built environment and health.
